# First report of the emerging pathogenic yeast *Candida palmioleophila* in commercially relevant fish from southeastern Brazil

**DOI:** 10.3389/ffunb.2025.1545572

**Published:** 2025-02-24

**Authors:** Manoel M. E. Oliveira, Viviane Felix Moraes Lima, Gisela Lara da Costa, Barbara de Oliveira Baptista, Julia Auad Augusto, Rachel Ann Hauser-Davis

**Affiliations:** ^1^ Laboratory of Taxonomy, Biochemistry and Bioprospecting of Fungi, Oswaldo Cruz Institute, Oswaldo Cruz Foundation, Rio de Janeiro, Brazil; ^2^ Laboratório de Avaliação e Promoção da Saúde Ambiental, Instituto Oswaldo Cruz, Fundação Oswaldo Cruz, Rio de Janeiro, Brazil; ^3^ Laboratório de Pesquisa em Malária, Instituto Oswaldo Cruz, Fundação Oswaldo Cruz, Rio de Janeiro, Brazil

**Keywords:** yeast, commercial fisheries, Sciaenidae, One Health, public health surveillance

## Abstract

The emerging fungal pathogen *Candida palmioleophila* (*C. palmioleophila*) has been increasingly detected in environmental and animal samples, although studies in this regard are still scarce, especially in fisheries contexts. This study reports the first-time detection of *C. palmioleophila* in a commercially relevant fish species belonging to the Sciaenidae family (*Cynoscion* sp.), indicating its potential emergence as a pathogen in Brazil. We applied CHROMagar Candida Plus medium identification associated to Matrix-Assisted Laser Desorption/Ionization Time-of-Flight Mass Spectrometry (MALDI-TOF MS) for the identification of *C. palmioleophila* isolates. Although only one fish specimen was shown to be contaminated by *C. palmioleophila*, this study provides the first evidence of this yeast circulating in commercially relevant fish species in Brazil, highlighting the potential risks associated with this emerging pathogen.

## Introduction

Fungal growth is strongly influenced by abiotic factors such as salinity, light, temperature, sediment conditions, and chemical pollution, all of which are being dramatically altered by the effects of climate change (De Crecy et al., 2009; Casadevall, 2012; Jones et al., 2022). Although the estimated number of marine fungal pathogens exceeds 10,000 species (Jones, 2011), studies investigating their impact remain relatively limited despite their growing importance. An increased occurrence and severity of diseases in marine animals, particularly those caused by fungi, has been observed in recent years (Garcia-Solache and Casadevall, 2010). These diseases can have significant ecological and economic consequences, affecting the health of marine organisms and potentially impacting fisheries and aquaculture industries. However, there is still a substantial gap in research on fungal pathogens for many commercially relevant species, leaving critical aspects of their epidemiology, host-pathogen interactions, and ecological implications poorly understood. This lack of knowledge highlights the urgent need for more comprehensive studies to assess the role of fungi in marine ecosystems and their potential threats to biodiversity and food security.

Fisheries production in southeastern Brazil, particularly in the states of São Paulo, Rio de Janeiro, Espírito Santo, and Paraná, plays a significant role in the country’s economy, both for local consumption and international trade. This region is known for its diverse and productive marine and freshwater ecosystems, which support a variety of commercial fishing activities. *Cynoscion* spp. is a marine group of species distributed across the western Atlantic Ocean, ranging from Panama to southeastern Brazil (Cervigón et al., 1993) and typically found in shallow coastal habitats, estuaries, and mangrove areas, often associated with sandy or muddy substrates, holding high economic value in Brazil (De Brito et al., 2015).

Recent studies have highlighted the growing interest in understanding fish microbiomes through the lens of the One Health approach, which emphasizes the interconnectedness of human, animal, and environmental health in monitoring and controlling the spread of zoonotic and emerging pathogens (Scheifler et al., 2023). This integrative perspective has brought attention to the occurrence of emerging fungal pathogens in both bony and cartilaginous fish species, with recent reports documenting their presence and potential implications (Peixoto-Rodrigues et al., 2023). Despite these advances, research on microbial communities in fish remains limited, particularly in Brazil. Most available studies focus on bacterial communities (Ayulo et al., 1994; Cardoso et al., 2024), while investigations into fungal communities, including yeasts, are scarce and relatively recent (Peixoto-Rodrigues et al., 2023; Oliveira et al., 2023). This data scarcity highlights the urgent need for further research to better understand the composition, dynamics, and role of yeast and fungal communities in fish health and food safety contexts, particularly given the high consumption of fish such as *Cynoscion* spp. in Brazil. In this sense, *Candida palmioleophila* (*C. palmioleophila*) is an ascomycetous yeast initially isolated from soil but later identified as an emerging human pathogen associated with catheter-related fungemia and multidrug resistance. It has been detected in diverse environments, including soil, water, agricultural settings, marine ecosystems, and wildlife, highlighting its relevance within a One Health framework, although studies are still scarce concerning this fungi species.

To address the gap concerning fungal communities in fish health and food safety contexts, the present study aimed to conduct a non-targeted yeast screening of the cloacal microbiota of *Cynoscion* spp. specimens sampled from Rio de Janeiro, Southeastern Brazil. A non-targeted approach offers several advantages, for example, enabling the comprehensive identification of a wide range of yeast species, including those that are not initially suspected, providing a more complete understanding of microbial diversity. It also allows for the discovery of novel or rare species, including emerging pathogens or beneficial organisms that might be missed in targeted methods and provides a holistic view of microbial communities, shedding light on species interactions across ecosystems. Additionally, non-targeted screening reduces bias by not preselecting species, ensuring that important organisms are not overlooked. Its flexibility makes it suitable for diverse research contexts, from natural ecosystems to clinical or industrial settings. This is the first study to examine the fungal microbiota of this species in southeastern Brazil, providing a valuable baseline report that could contribute to food safety and broader microbiological research.

## Methodology

*Cynoscion* spp. specimens (n=10, five from a dry season sampling and five from a wet season sampling in 2023) were captured by artisanal fishers in a fisher colony of Barra da Tijuca, in Rio de Janeiro, southeastern Brazil. Samplings were conducted on recently deceased animals, with no need for any previous authorization as they are sold for consumption. Cloacal swabs were obtained by inserting sterile swabs into the entire cloacal cavity of each animal for 2 to 3 seconds. The swabs were then placed in 2 mL microtubes containing a 0.9% saline solution. The samples were transported to the laboratory within 30 minutes of collection and stored at 4°C until further analysis.

At the laboratory, samples were streaked onto Sabouraud Dextrose Agar (SDA) and incubated at 30°C for 48 hours to allow for morphological assessments. Colonies presenting distinct macromorphological characteristics on SDA were subcultured onto CHROMagar Candida (BD Difco) and CHROMagar Candida Plus (CHROMagar™) at 37°C. Colony characteristics on these selective media were then interpreted following the manufacturer’s guidelines to confirm the presence of different yeast species.

Species-level identification of the fungal isolates was performed using a polyphasic taxonomy approach, combining morphological and phenotypic analyses with molecular techniques, namely MALDI-TOF MS and ITS region sequencing, as described by Pinto et al. (2022) and Oliveira et al. (2023).

Fungal identification via MALDI-TOF MS followed by Oliveira et al. (2023). Briefly, about 10⁶ yeast cells (~1 μg) were transferred from the culture plates into 500 μL tubes containing 20 μL of 70% formic acid (v/v) and mixed with 10 μL of acetonitrile. Subsequently, 1 μL of this mixture was spotted onto a stainless steel MALDI-TOF MS plate (Bruker, UK), covered with 1 μL of an α-cyano-4-hydroxycinnamic acid matrix solution (CHCA, Fluka, Switzerland) and air-dried at room temperature prior to spectra acquisition. Each sample was analyzed in triplicate. Identification scores were expressed as log values ranging from 0 to 3, with values ≥1.7 considered reliable for genus-level identification and ≥2.0 for species-level identification (Pinto et al., 2022).

For ITS region sequencing, performed at the Fundação Oswaldo Cruz (PDTIS/FIOCRUZ) Sequencing Platform (Rio de Janeiro, Brazil), colony PCR was performed as outlined by Corrêa-Moreira et al. (2024). Yeast colonies grown on SDA plates at 30°C for 48 h were used as the DNA source. A small portion of each isolated colony was transferred with a micropipette tip directly into PCR tubes as the DNA template. Cells were lysed by heating in a microwave for 90 seconds, followed by immediate cooling on ice to prevent DNA degradation. PCR amplification was carried out in a 50 μL reaction mixture containing 25 ng of genomic DNA, 10 pmol of universal fungal primers ITS1 (CGTAGGTGAACCTGCGG) and ITS4 (TCCTCCGCTTATTGATATGC), using an annealing temperature of 58°C in a 96-well thermocycler (Applied Biosystems, Thermo Fisher Scientific). The amplified products were purified with a QIAquick^®^ PCR Purification Kit (QIAGEN^®^) according to the manufacturer’s protocol.

The resulting sequences were edited using CodonCodeAligner v. 9.0.2 software and compared to NCBI GenBank entries via the Basic Local Alignment Search Tool (BLAST). A phylogenetic analysis was conducted employing the neighbor-joining algorithm of Saitou and Nei (1987) using the MEGA X software (https://www.megasoftware.net), with a bootstrap replication (1000 replicates) to assess tree robustness. Evolutionary distances were computed using the Maximum Composite Likelihood method (Tamura et al., 2004).

## Results and discussion

**Figure 1:**
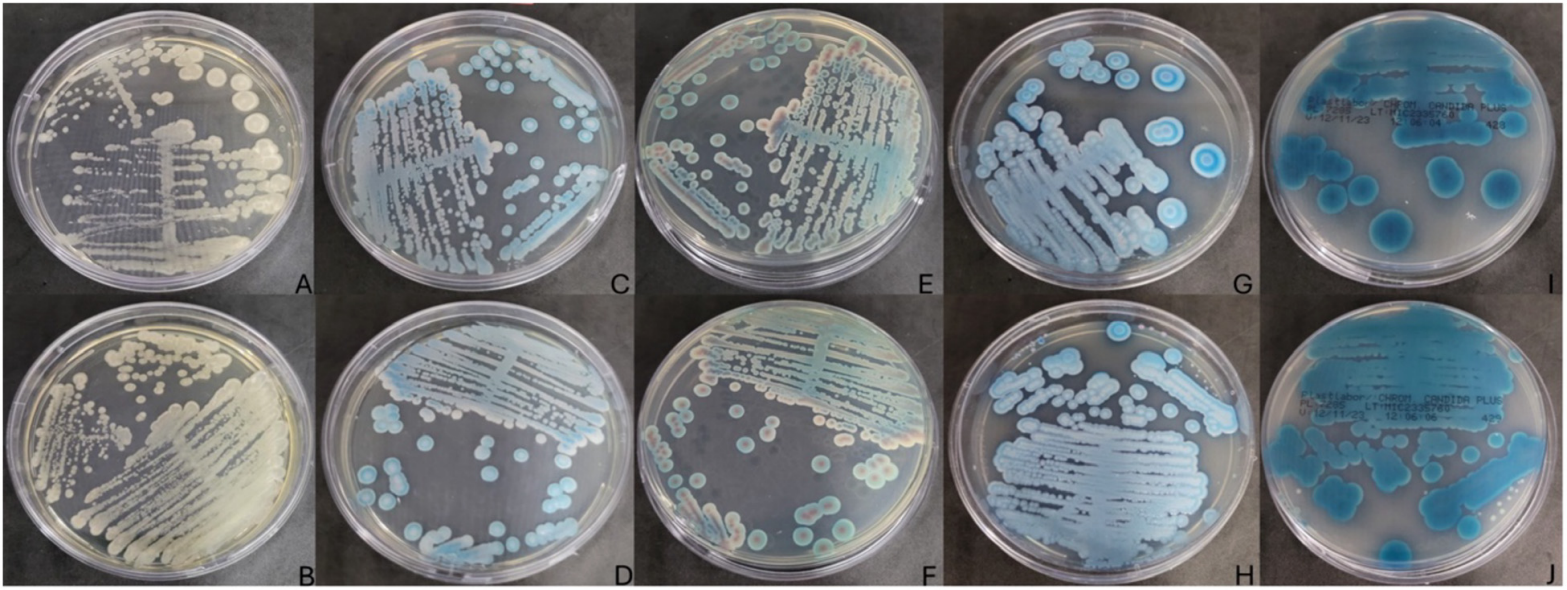
Fungal growth in SDA Medium (BD Difco) incubated at 30°C for 48 hours, **(A)** fish sample IOC/RPPM04/2, **(B)** fish sample IOC/RPPM04/3. Fungal growth in BDTM CHROMagar™ Candida Medium (BD Difco) **(C, E)**, fish samples IOC/RPPM04/2 and IOC/RPPM04/3 **(D, F)**. CHROMagar Candida PlusTM (CHROMagar, France) at 37°C for 48 hours **(G, I)**, fish samples IOC/RPPM04/2 and IOC/RPPM04/3 **(H, J)**. The isolate numbers are linked to the specific fish from which each yeast was sourced, where IOC indicates the standard culture collection acronym, while RPPM04 followed by a number refer to the fish from which the yeasts were isolated.

Two distinct fungal colonies were observed in one *Cynoscion* spp. specimen sampled in the rainy season of 2023, among a total of 10 individuals, with growth detected on SDA medium during the fungal screening process (**Figures 1A, B**). These were the only two yeast isolates observed in the study.

These colonies were subsequently subcultured onto CHROMagar Candida (BD Difco) and CHROMagar Candida Plus (CHROMagarTM) for further identification (**Figures 1C–J**).

Distinct color patterns were observed on the chromogenic medium, namely turquoise, rose, and white (**Figures 1C–J**). According to Jensen and Arendrup (2011) and Costa et al. (2023), turquoise or rose colorations are indicative of *C. palmioleophila*.

The morphological and phenotypic characterization of the isolates was then complemented by MALDI-TOF MS and ITS region sequencing analyses for yeast species identification (Pinto et al., 2022; Oliveira et al., 2023).

Isolates were identified at the species level employing a Microflex LT mass spectrometer (Bruker Daltonics) and the Biotyper™ 3.1 software (Bruker Daltonics) using the MALDI-TOF MS Bruker database, where they were classified as *C. palmioleophila*, with score values of 2.12 IOC/RPPM04/2 and 2.17 IOC/RPPM04/3.

**Figure 2:**
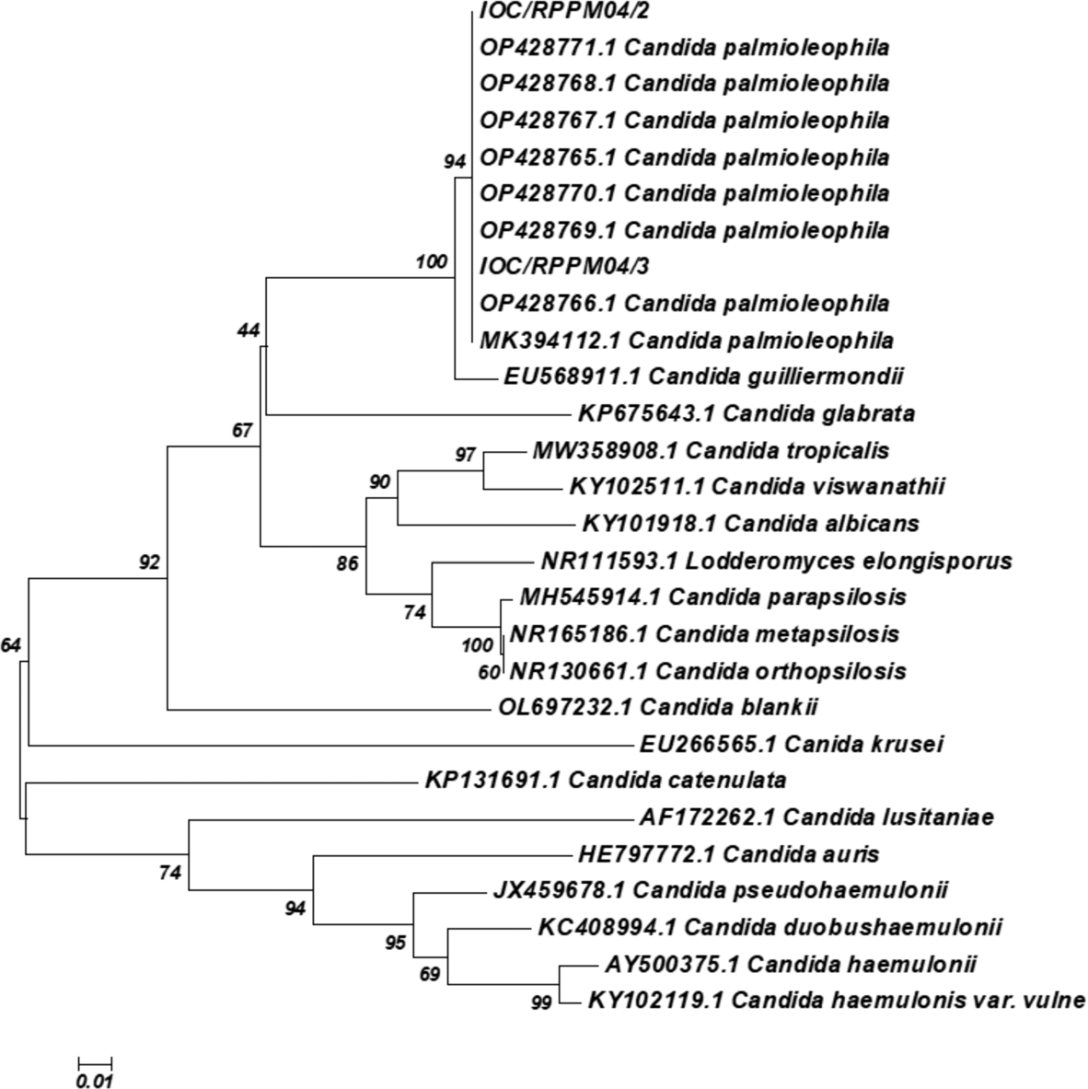
Phylogenetic relationships between the isolate of samples with reference strains of *Candida* spp. inferred from ITS sequences by the Neighbor-Joining method (Saitou and Nei, 1987). The optimal tree is shown. The percentages of replicate trees in which the associated taxa clustered together in the bootstrap test (1000 replicates) are shown next to the branches (Felsenstein, 1985). The evolutionary distances were computed using the Maximum Composite Likelihood method (Tamura et al., 2004) and are in the units of the number of base substitutions per site. This analysis involved 28 nucleotide sequences. A total of 218 positions were obtained in the final dataset. Evolutionary analyses were conducted using the MEGA X software (Tamura et al., 2007).

Following editing using the CodonCodeAligner 9.0.2 software and comparison to sequences deposited at the NCBI/GenBank database using BLAST. The ITS sequence analysis confirmed the identification of both isolates as *C. palmioleophila*, with comparisons to available ITS sequences from GenBank (*C. palmioleophila* MK394112.1 clinical sample and OP428765.1 to OP428771.1 environmental samples) (**Figure 2**). The ITS sequences of these isolates have been deposited in GenBank under accession numbers PQ760050(RPPM04/02) and PQ760051(IOC/RPPM04/03).

The MALDI-TOF MS and partial ITS region sequencing results showed 100% agreement, confirming species identification by both methods.

The MALDI-TOF MS technique has been recently introduced for identifying fungal species across various genera, including isolates obtained from animals (Jensen and Arendrup, 2011; Oliveira et al., 2015; Valero et al., 2018), comprising a valuable tool in fungal identification, including rare species (Costa et al., 2023). From an identification perspective, CHROMagar Candida medium can be a useful assisting tool, as demonstrated by Costa et al. (2023), who reported the establishment of a color standard for the emerging species *C. palmioleophila*.

*Candida palmioleophila*, an ascomycetous yeast, was first isolated from soil by Nakase et al. (1988) and later identified as the causative agent of catheter-related fungemia (Sugita et al., 1999). Although it is a rare human pathogen, the frequency of infections *C. palmioleophila* has increased, and it is now considered an emerging species of concern in European countries such as Denmark (Arendrup et al., 2010) and Italy (Pierantoni et al., 2020), due to several cases of associated drug resistance (Datta et al., 2015; Mroczynska and Brillowska-Dabrowska, 2019; Pierantoni et al., 2020; Lavergne et al., 2023). This includes resistance to fluconazole (Xiao et al., 2018; Costa et al., 2023), with one fatal case reported in China (Wu et al., 2023). In that case, despite the yeast’s dose-dependent sensitivity to fluconazole, the CDR1 and MDR1 genes, both associated with azole resistance, were detected (Wu et al., 2023). Multidrug resistance has also been noted, including to other azoles and echinocandins (Stavrou et al., 2020).

This yeast has been detected in various environments, highlighting its relevance within a One Health framework, which connects environmental, animal, and human health. For example, it has been observed in fruits, soil, water, and farmers in Taiwanese orchards, indicating a potential role in environmental reservoirs of pathogenic yeasts in agricultural settings (Tseng et al., 2024). Furthermore, it has also been detected in different marine ecosystems, *i.e.*, swamps and sediment from continental platforms in India (PrasannaKummar et al., 2020); oligotrophic hypersaline coastal waters in the Arabian Gulf (Fotedar et al., 2022); a hydrographic basin in the state of Minas Gerais, Brazil, notably during the dry season (Medeiros et al., 2012), as well as in hospital wastewaters in Mexico (Treviño-Trejo et al., 2023) and wastewaters in the city of Niterói, also in Brazil, during the COVID-19 pandemic (Costa et al., 2023). It has also been detected in wildlife representatives, such as free-ranging Magellanic penguins (*Spheniscus magellanicus*) (Ewbank et al., 2021), representing the first report of candidiasis caused by *C. palmioleophila* in penguins globally and suggesting the species’ susceptibility to fungal infections in the wild, indicating potential links between fungal diseases and factors such as poor body condition, impaired immunity, and migration-related stressors. It has also been identified in two cultured marine Chilean fishes, the red cusk eel (*Genypterus chilensis*) and the palm ruff (*Seriolella violacea*) (Valderrama et al., 2021), although no further fish detections of this fungus have been reported to date, indicating the importance of this assessment in a One Health context.

The increasing environmental detection of *C. palmioleophila*, including in wastewater which is often discharged into various aquatic environments, without treatment, as well as cases of infection and emerging resistance profile emphasizes the importance of monitoring fungal pathogens in aquatic environments. In this case, fish often play a role as vectors for the transmission of resistant pathogens to humans (Gozlan et al., 2014; Al Sulivany et al., 2024) and the consumption of contaminated fish could increase the risk of human exposure to drug-resistant *Candida* strains, highlighting the need for continuous surveillance and management of fungal resistance in both environmental and public health contexts. In this sense, fish serve as a primary protein source in Brazil, and contamination by opportunistic pathogens like *C. palmioleaphila* raises food safety concerns. Even though only one fish specimen was shown to be contaminated by *C. palmioleophila*, our study provides the first evidence of this yeast circulating in commercially relevant fish species in Brazil, highlighting the potential risks associated with this emerging pathogen. The yeast-positive specimen was sampled in the rainy season of 2023, in contrast to the detection of *C. palmioleophila* during the dry season in a hydrographic basin in the state of Minas Gerais, Brazil, which may indicate seasonality variations that should be further investigated.

While it is true that *C. palmioleophila* is commonly found in various environmental isolates, our study indicated its presence in fish microbiomes, an area that has been relatively underexplored in previous research. This suggests that the environment the analyzed fish species inhabits may provide a unique habitat for certain yeasts, including *C. palmioleophila*, which could serve not only as a potential pathogen but also as a valuable indicator of environmental health and microbial diversity within aquatic ecosystems. However, further investigations are needed to clarify whether the analyzed fish act merely as a passive environmental sieve, a true reservoir, or a potential disease vector. The fisheries environment, in this regard, plays a particularly significant role, as it serves as a dynamic interface between aquatic organisms, their pathogens, and human activities, comprising an essential area for monitoring public health risks and ecosystem health. The presence of *C. palmioleophila* in these environments could, therefore, be an important marker of environmental conditions, potentially reflecting ecosystem shifts that affect both aquatic life and human health. These findings, therefore, further indicate the relevance of the One Health approach since, by incorporating environmental surveillance, as demonstrated in our study, the One Health strategy enables early detection and risk assessment of emerging yeast pathogens, facilitating timely intervention to prevent their spread across different ecosystems, including those shared by wildlife, domestic animals, and humans. This integrated approach is critical for mitigating public health threats, particularly in countries like Brazil, where environmental contamination and interactions between wildlife, livestock, and humans are closely linked.

We, however, acknowledge that the current study has limitations, including a relatively small sample size (n=10) and a narrow geographical scope, which may hinder a comprehensive understanding of yeast prevalence in *Cynoscion* spp. Additionally, the absence of other yeasts in the investigated fish samples suggests either a specific ecological association between *C. palmioleophila* and *Cynoscion* spp. or limitations in the study’s sampling or detection methods. Finally, we highlight *C. palmioleophila* as a potential emerging pathogen, this characterization is constrained by the absence of antifungal susceptibility testing and the lack of clinical data from Brazil to substantiate its emergence. Future assessments will address these limitations by expanding the sampling to include a broader, regionally diverse range of specimens and incorporating antifungal susceptibility testing. If the observed isolation ratio (e.g., 10%) is confirmed in larger datasets, this would provide stronger evidence to support the claim of emergence. In addition, while antifungal susceptibility testing was not included in the current study we recognize its significance and plan to conduct antifungal susceptibility testing in future assessments to provide important insights into the potential risks posed by *C. palmioleophila* isolates. These steps will be critical to substantiate our findings and further investigate the potential role of *C. palmioleophila* in fish health and its implications for food safety.

One a side note, it is important to note that *C. palmioleophila* has previously been misidentified as *C. famata*. One key differentiating characteristic between these two species is the ability to hydrolyze esculin. Thus, the esculin hydrolysis test should be incorporated in future studies as an additional confirmatory step for yeast species identification, further substantiating the accuracy and effectiveness of the MALDI-TOF MS identification method.

## Conclusion

This study comprises a first-time report of *C. palmioleophila* from a commercially relevant fish species in Brazil, highlighting this yeast as a potential emerging pathogen in the country, even more so due to climate change effects, with implications for both animal and human health. Furthermore, the MALDI-TOF MS technique was proven adequate for yeast identification, providing a faster, more effective, and cost-efficient method compared to conventional phenotypic approaches and genetic sequencing.

## Data Availability

The datasets presented in this study can be found in online repositories. The names of the repository/repositories and accession number(s) can be found in the article/supplementary material.
